# Supplementary material for: Language statistical learning responds to reinforcement learning principles rooted in the striatum
Source: PLoS Biol. 2021 Sep 7;19(9):e3001119. doi: 10.1371/journal.pbio.3001119 (PMC8448350; doi:10.1371/journal.pbio.3001119)
Supplement: S1 Fig — Plot of (A) behavioral group and (B) fMRI group participants’ mean RTs (blue) against the RW model’s estimates of the development of predictions over learning (red; inverted as 1-P(A) before averaging and z-scoring for display purposes). Vertical bars are the SD. RTs were initially transformed (Materials and methods) and are plotted with the model prediction estimates in z-score values. P(A) = RW model’s predictions from the initial word (A) of the dependencies. Data used to generate S1 Fig can be found in S3 Data. fMRI, functional magnetic resonance imaging; RT, reaction time; RW, Rescorla-Wagner. (DOCX) [file pbio.3001119.s001.docx]

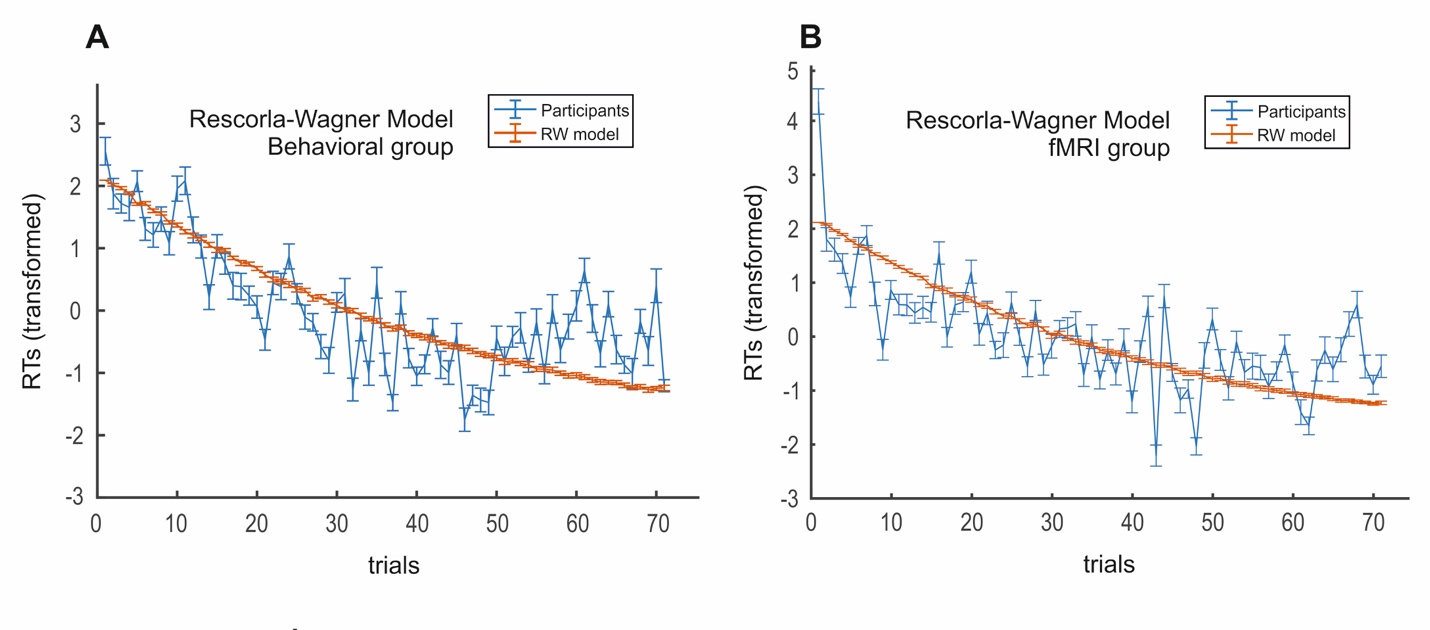


**S1 Fig.** Plot of **(A)** Behavioral group and **(B)** fMRI group participants’ mean **reaction times** (blue) **against the** **Rescorla-Wagner model’s estimates** of the development of predictions over learning (red; inverted as 1-*P*(A) before averaging and z-scoring for display purposes). Vertical bars are the SD. RTs = reaction times. Reaction times were initially transformed (Materials and methods) and are plotted with the model prediction estimates in z-score values. *P*(A) = Rescorla-Wagner model’s predictions from the initial word (A) of the dependencies. Data used to generate S1 Fig can be found in S3 Data.
