# Supplementary material for: Language statistical learning responds to reinforcement learning principles rooted in the striatum
Source: PLoS Biol. 2021 Sep 7;19(9):e3001119. doi: 10.1371/journal.pbio.3001119 (PMC8448350; doi:10.1371/journal.pbio.3001119)
Supplement: S2 Fig — Activity in the basal ganglia (bilateral caudate nuclei, putamen, and ventral striatum; see S2 Table) was modulated by the trial-by-trial development of predictions (P(A)) as estimated by the TD model. Results are reported for clusters FWE-corrected at p < 0.001 at the cluster level (minimum cluster size = 20). Neurological convention is used with MNI coordinates shown at the bottom right of each slice. Data used to generate S2 Fig can be found in http://identifiers.org/neurovault.collection:10421. FWE, family-wise error; NAD, nonadjacent dependency; STG, superior temporal gyrus; TD, temporal difference. (DOCX) [file pbio.3001119.s002.docx]

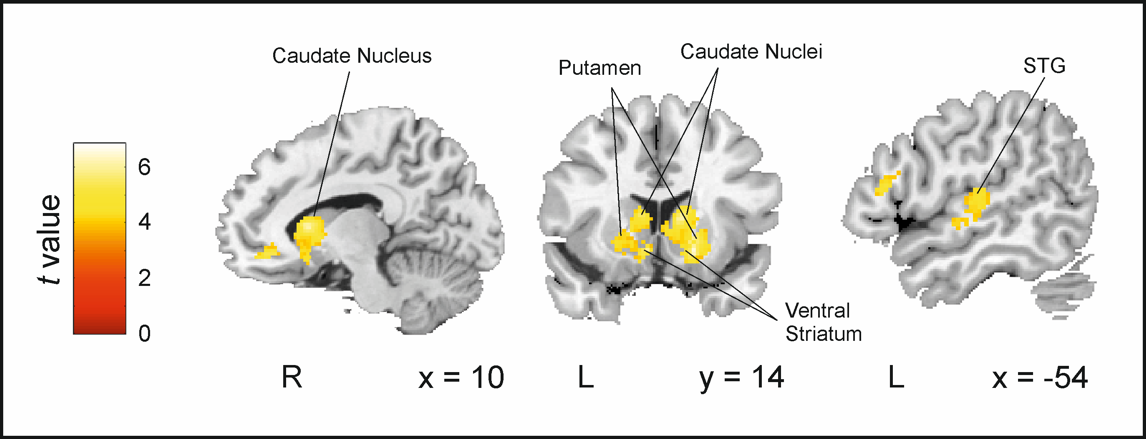


**S2 Fig. Brain regions related to changes in the predictive value of the initial word of each phrase in the NADs block (*P*(A)) versus changes in the predictive value of the initial word of each phrase (*P*(X1)) in the Random block (i.e., *P*(A)-modulated NADs block vs. *P*(X1)-modulated Random block).** Activity in the Basal Ganglia (bilateral caudate nuclei, putamen, and ventral striatum; see S2 Table) was modulated the trial-by-trial development of predictions (*P*(A)) as estimated by the TD model. Results are reported for clusters FWE-corrected at *p* < 0.001 at the cluster level (minimum cluster size = 20). Neurological convention is used with MNI coordinates shown at the bottom right of each slice. Data used to generate S2 Fig can be found in http://identifiers.org/neurovault.collection:10421.
