# Supplementary material for: Language statistical learning responds to reinforcement learning principles rooted in the striatum
Source: PLoS Biol. 2021 Sep 7;19(9):e3001119. doi: 10.1371/journal.pbio.3001119 (PMC8448350; doi:10.1371/journal.pbio.3001119)
Supplement: S3 Fig — Subtracting the activity for the Random block modulated by participants’ RTs from the P(A)-modulated NADs block activity had virtually no effect on basal ganglia activity estimates (see S3 Table). Significant activity centered on the caudate nuclei and the right putamen. Results are reported at a p < 0.001 FWE-corrected threshold at the cluster level with 20 voxels of minimum cluster extent. Neurological convention is used with MNI coordinates shown at the bottom right of each slice. Data used to generate S3 Fig can be found in http://identifiers.org/neurovault.collection:10421. NAD, nonadjacent dependency; RT, reaction time. (DOCX) [file pbio.3001119.s003.docx]

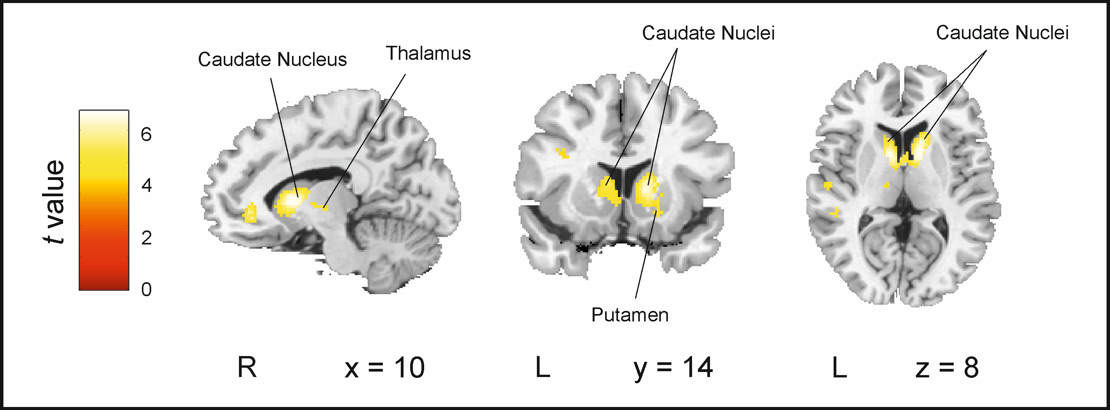


**S3 Fig. Brain regions related to changes in the predictive value of the initial word of each phrase in the NADs block (*P*(A)) controlling for overt motor response activity in the Random block (i.e., *P*(A)-modulated NADs block vs. RT-modulated Random block).** Subtracting the activity for the Random block modulated by participants’ reaction times from the *P*(A)-modulated NADs block activity had virtually no effect on Basal Ganglia activity estimates (see S3 Table). Significant activity centred on the caudate nuclei and the right putamen. Results are reported at a *p* < 0.001 FWE-corrected threshold at the cluster level with 20 voxels of minimum cluster extent. Neurological convention is used with MNI coordinates shown at the bottom right of each slice**.** Data used to generate S3 Fig can be found in http://identifiers.org/neurovault.collection:10421.
