# Supplementary material for: Language statistical learning responds to reinforcement learning principles rooted in the striatum
Source: PLoS Biol. 2021 Sep 7;19(9):e3001119. doi: 10.1371/journal.pbio.3001119 (PMC8448350; doi:10.1371/journal.pbio.3001119)
Supplement: S4 Fig — Significant activations by the contrast between P(A)-modulated NADs block and RT-modulated NADs block activity (see also S4 Table) were found in a widespread left-lateralized network of areas, including a large portion of the IFG, parts of the pre- and post-CG, and of the STG in and around the left auditory cortex. Interestingly, bilateral caudate nuclei were also statistically significant along with a small portion of the thalamus. Results are reported for clusters FWE-corrected at p < 0.001 at the cluster level (minimum cluster size = 20). Neurological convention is used with MNI coordinates shown at the bottom right of each slice. Data used to generate S4 Fig can be found in http://identifiers.org/neurovault.collection:10421. CG, central gyrus; FWE, family-wise error; IFG, inferior frontal gyrus; NAD, nonadjacent dependency; RT, reaction time; STG, superior temporal gyrus; TTG, transverse temporal gyrus. (DOCX) [file pbio.3001119.s004.docx]

**
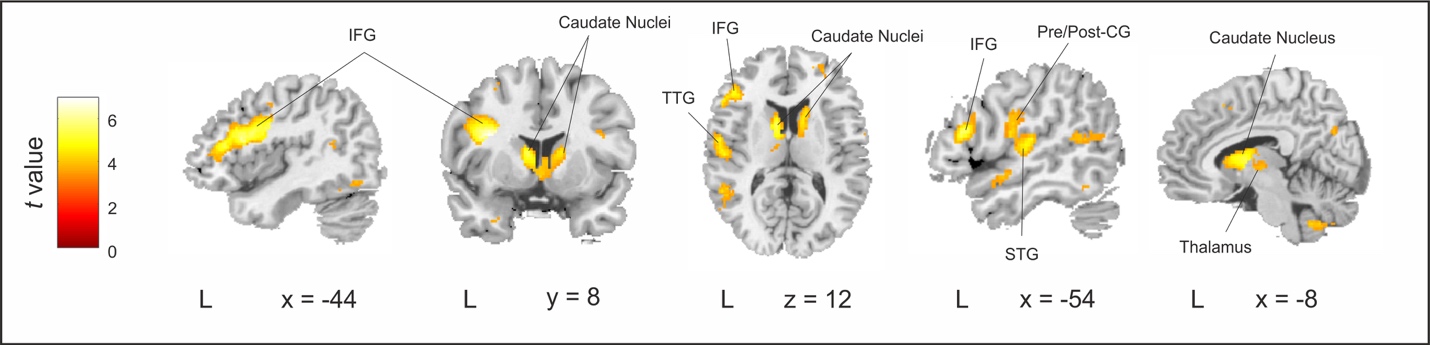
**

**S4 Fig. Brain regions related to changes in the predictive value of the initial word of each phrase in the NADs block (*P*(A)) controlling for overt motor response activity in the NADs block (i.e., *P*(A)-modulated NADs block vs. RT-modulated NADs block).** Significant activations by the contrast between *P*(A)-modulated NADs block and RT-modulated NADs block activity (see also S4 Table) were found in a widespread left-lateralized network of areas, including a large portion of the inferior frontal gyrus, parts of the pre and post central gyri, and of the superior temporal gyrus in and around the left auditory cortex. Interestingly, bilateral caudate nuclei were also statistically significant along with a small portion of the thalamus. Results are reported for clusters FWE-corrected at *p* < 0.001 at the cluster level (minimum cluster size = 20). Neurological convention is used with MNI coordinates shown at the bottom right of each slice. Data used to generate S4 Fig can be found in http://identifiers.org/neurovault.collection:10421.
