# Supplementary material for: Language statistical learning responds to reinforcement learning principles rooted in the striatum
Source: PLoS Biol. 2021 Sep 7;19(9):e3001119. doi: 10.1371/journal.pbio.3001119 (PMC8448350; doi:10.1371/journal.pbio.3001119)
Supplement: S5 Fig — Model fit to each participant’s fMRI data and vectors used to compute the specified contrasts. fMRI, functional magnetic resonance imaging; NAD, nonadjacent dependency. (DOCX) [file pbio.3001119.s005.docx]

**
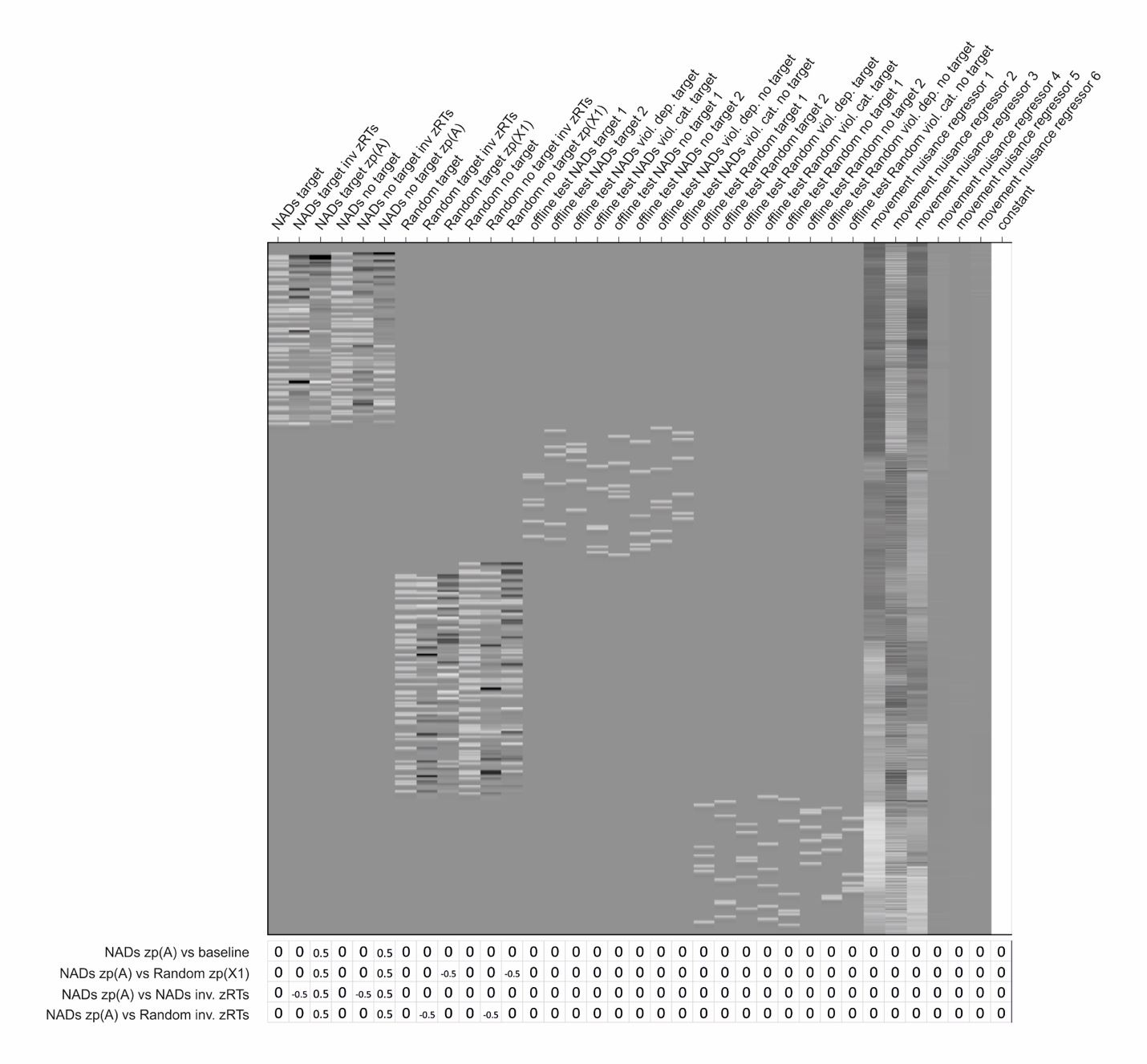
**

**S5 Fig. fMRI first-level model and contrasts. A.** Model fit to each subject’s fMRI data and vectors used to compute the specified first-level contrasts.
