# Supplementary material for: Language statistical learning responds to reinforcement learning principles rooted in the striatum
Source: PLoS Biol. 2021 Sep 7;19(9):e3001119. doi: 10.1371/journal.pbio.3001119 (PMC8448350; doi:10.1371/journal.pbio.3001119)
Supplement: S1 Table — Group-level fMRI local maxima for the P(A)–modulated NADs block against implicit baseline contrast (see also red-yellow regions in Fig 4, main text). Results are reported for clusters FWE-corrected at p < 0.001 at the cluster level (minimum cluster size = 20). MNI coordinates were used. BA, Brodmann area; fMRI, functional magnetic resonance imaging; FWE, family-wise error; NAD, nonadjacent dependency. (DOCX) [file pbio.3001119.s006.docx]

**S1 Table. Whole brain fMRI activity for the NADs *P*(A)-modulated activity vs. implicit baseline contrast.** Group-level fMRI local maxima for the *P*(A)–modulated NADs block against implicit baseline contrast (see also red-yellow regions in Fig 4, main text). Results are reported for clusters FWE-corrected at *p* < 0.001 at the cluster level (minimum cluster size = 20). MNI coordinates were used. BA, Brodmann Area.

| Anatomical area | Coordinates | Cluster Size | *t*-value |
| --- | --- | --- | --- |
| Right Caudate  Left Caudate  Right Putamen  Left Putamen | 18 22 0 | 1760 | 6.03 |
| Right Middle Occipital Cortex  Right Inferior Occipital Cortex  Right Middle Temporal Gyrus | 38 -84 18 | 687 | 5.66 |
| Left Superior Temporal Gyrus (BA41/42)  Left Transverse Temporal Gyrus  Left Insula / Rolandic Operculum  Left Precentral Gyrus  Left Postcentral Gyrus  Left Heschl’s Gyrus  Left Middle Temporal Gyrus | -56 -14 10 | 1078 | 4.64 |
