# Supplementary material for: Language statistical learning responds to reinforcement learning principles rooted in the striatum
Source: PLoS Biol. 2021 Sep 7;19(9):e3001119. doi: 10.1371/journal.pbio.3001119 (PMC8448350; doi:10.1371/journal.pbio.3001119)
Supplement: S2 Table — Group-level fMRI local maxima for the P(A)–modulated NADs block minus P(X1)–modulated Random block contrast (see also red-yellow regions in S2 Fig). Results are reported for clusters FWE-corrected at p < 0.001 at the cluster level (minimum cluster size = 20). MNI coordinates were used. BA, Brodmann area; fMRI, functional magnetic resonance imaging; FWE, family-wise error; NAD, nonadjacent dependency. (DOCX) [file pbio.3001119.s007.docx]

**S2 Table. Whole brain fMRI activity for the *P*(A)-modulated NADs block vs. *P*(X1)-modulated Random block contrast.** Group-level fMRI local maxima for the *P*(A)–modulated NADs block minus *P*(X1)–modulated Random block contrast (see also red-yellow regions in S2 Fig). Results are reported for clusters FWE-corrected at *p* < 0.001 at the cluster level (minimum cluster size = 20). MNI coordinates were used. BA, Brodmann Area.

| Anatomical area | Coordinates | Cluster Size | *t*-value |
| --- | --- | --- | --- |
| Right Caudate  Left Caudate  Left Putamen  Right Putamen | 16 20 8 | 1239 | 6.03 |
| Right Middle Occipital Cortex  Right Middle Temporal Gyrus | 36 -84 10 | 384 | 5.24 |
| Left Superior Temporal Gyrus (BA41/42)  Left Transverse Temporal Gyrus | -48 -28 10 | 387 | 4.82 |
