# Supplementary material for: Language statistical learning responds to reinforcement learning principles rooted in the striatum
Source: PLoS Biol. 2021 Sep 7;19(9):e3001119. doi: 10.1371/journal.pbio.3001119 (PMC8448350; doi:10.1371/journal.pbio.3001119)
Supplement: S3 Table — Group-level fMRI local maxima for the P(A)-modulated NADs block minus RT-modulated Random block contrast (see also red-yellow regions in S3 Fig). Results are reported at a p < 0.001 FWE-corrected threshold at the cluster level with 20 voxels of minimum cluster extent. MNI coordinates were used. BA, Brodmann area; fMRI, functional magnetic resonance imaging; FWE, family-wise error; NAD, nonadjacent dependency; RT, reaction time. (DOCX) [file pbio.3001119.s008.docx]

**S3 Table. Whole brain fMRI activity for the *P*(A)-modulated NADs block vs. RT-modulated Random block contrast.** Group-level fMRI local maxima for the *P*(A)-modulated NADs block minus RT-modulated Random block contrast (see also red-yellow regions in S3 Fig). Results are reported at a *p* < 0.001 FWE-corrected threshold at the cluster level with 20 voxels of minimum cluster extent. MNI coordinates were used. BA, Brodmann Area.

| Anatomical area | Coordinates | Cluster Size | *t*-value |
| --- | --- | --- | --- |
| Right Caudate  Left Caudate  Thalamus  Right Putamen | 10 10 6 | 1058 | 5.60 |
