# Supplementary material for: Language statistical learning responds to reinforcement learning principles rooted in the striatum
Source: PLoS Biol. 2021 Sep 7;19(9):e3001119. doi: 10.1371/journal.pbio.3001119 (PMC8448350; doi:10.1371/journal.pbio.3001119)
Supplement: S4 Table — Group-level fMRI local maxima for the P(A)-modulated NADs block minus RT-modulated NADs block contrast (see also red-yellow regions in S4 Fig). Results are reported for clusters FWE-corrected at p < 0.001 at the cluster level (minimum cluster size = 20). MNI coordinates were used. BA, Brodmann area; fMRI, functional magnetic resonance imaging; FWE, family-wise error; NAD, nonadjacent dependency; RT, reaction time. (DOCX) [file pbio.3001119.s009.docx]

**S4 Table. Whole brain fMRI activity for the *P*(A)-modulated NADs block vs. RT-modulated NADs block contrast.** Group-level fMRI local maxima for the *P*(A)-modulated NADs block minus RT-modulated NADs block contrast (see also red-yellow regions in S4 Fig). Results are reported for clusters FWE-corrected at *p* < 0.001 at the cluster level (minimum cluster size = 20). MNI coordinates were used. BA, Brodmann Area.

| Anatomical area | Coordinates | Cluster Size | *t*-value |
| --- | --- | --- | --- |
| Left Inferior Frontal Gyrus (Pars Triangularis)  Left Pre-Central Gyrus  Left Post-Central Gyrus  Left Inferior Frontal Gyrus (Pars Opercularis)  Left Middle Frontal Gyrus  Left Superior Temporal Gyrus (BA41/42)  Left Transverse Temporal Gyrus  Left Heschl’s Gyrus / Rolandic Operculum | -34 10 24 | 1796 | 6.95 |
| Left Caudate  Right Caudate  Left Thalamus  Right Thalamus | -10 6 10 | 962 | 5.48 |
| Left Middle Occipital Cortex  Left Angular Gyrus  Left Precuneus  Left Superior Occipital Cortex | -28 -52 40 | 369 | 4.96 |
