# Supplementary material for: Language statistical learning responds to reinforcement learning principles rooted in the striatum
Source: PLoS Biol. 2021 Sep 7;19(9):e3001119. doi: 10.1371/journal.pbio.3001119 (PMC8448350; doi:10.1371/journal.pbio.3001119)
Supplement: S5 Table — Group-level fMRI local maxima for the NADs block against Random block contrast covarying with the NADs Effect (see Fig 5). Results are reported for clusters FWE-corrected at p < 0.001 at the cluster level (minimum cluster size = 20). MNI coordinates were used. BA, Brodmann area; fMRI, functional magnetic resonance imaging; FWE, family-wise error; NAD, nonadjacent dependency; RT, reaction time. (DOCX) [file pbio.3001119.s010.docx]

**S5 Table. Whole brain fMRI for the NADs block vs. Random block activity covarying with the NADs effect contrast.** Group-level fMRI local maxima for the NADs block against Random block contrast covarying with the NADs Effect (see Fig 5). Results are reported for clusters FWE-corrected at *p* < 0.001 at the cluster level (minimum cluster size = 20). MNI coordinates were used. BA, Brodmann Area.

| Anatomical area | Coordinates | Cluster Size | *t*-value |
| --- | --- | --- | --- |
| Left Insula  Left Anterior Parahippocampal Gyrus  Left Amygdala  Left Putamen | -34 -16 -2 | 509 | 6.12 |
| Left Superior Temporal Gyrus  Left Pre-Central Gyrus  Left Post-Central Gyrus  Left Rolandic Operculum  Left Insula | -60 -30 12 | 398 | 5.41 |
