# Supplementary material for: Language statistical learning responds to reinforcement learning principles rooted in the striatum
Source: PLoS Biol. 2021 Sep 7;19(9):e3001119. doi: 10.1371/journal.pbio.3001119 (PMC8448350; doi:10.1371/journal.pbio.3001119)
Supplement: S1 Text — Results of the offline recognition test that participants performed after the online incidental NAD learning task. NAD, nonadjacent dependency. (DOCX) [file pbio.3001119.s011.docx]

**S1 Text. Offline Recognition Test**

Following each block, participants’ knowledge of the structures was assessed via a recognition test. Participants were presented with correct sentences (phrases that conformed the structures) and incorrect sentences (phrases that violated the structures). In half the trials, incorrect sentences consisted of violations of the A_C dependencies where A and C elements maintained their correct order within the phrase but belonged to different structures (i.e., A1xC2, A2xC1). In the other half, incorrect sentences contained order violations, where elements from category A and C in a given dependency swapped positions (i.e., C1xA1 and C2xA2). The complete offline test consisted in a total of 48 test phrases (24 per dependency). Participants were required to discriminate phrases that could belong to the previously heard language from phrases that could not by pressing the appropriate button. A maximum of 1500ms was allowed to respond, after which there was a jittered interval (1-3 secs.) before the next trial began. Participants’ ability to discriminate correct structure items from violations was assessed by computing *d* prime scores (d′) from their responses. For each participant, the proportion of hits (i.e., yes responses to correct phrases) and false alarms (i.e., yes responses to incorrect phrases) was used to calculate the d′ score. Hit and false alarm rates of zero or one were corrected according to Macmillan and Kaplan (1985). We computed two distinct d’ scores by using false alarms to 1) order violations (d’ Cat) and 2) to dependency violations (d’ Dep). These scores were then submitted to one-sample t-tests against 0 to determine statistical significance. After a NADs block, the test for the corresponding language’s NADs block was administered. After a Random block, the test for the corresponding language’s NADs block was also administered as a control. We expected learning of the language’s specific dependencies (significant d’ Dep) only in the NADs block but learning of positional information (significant d’ Cat) after both NADs and Random blocks since in both conditions the target word appeared always at the end of the phrase.


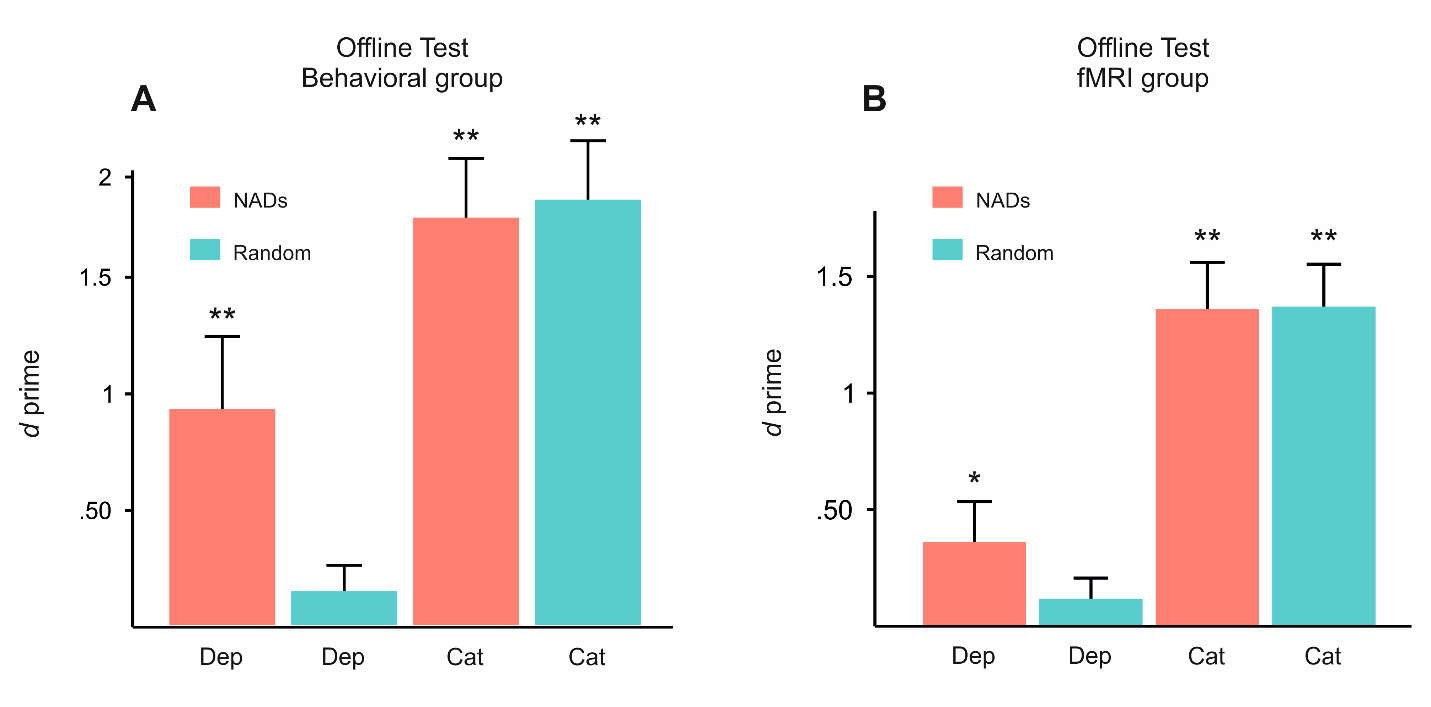


**Fig A.** Performance in the offline Recognition Test. **(A)** Behavioral group performance in the discrimination (*d* prime) between A_1_xC_1_ and A_1_xC_2_ (Dependency violations, Dep) and between A_1_xC_1_ and C_1_xA_1_ (Positional violations, Cat). **(B)** FMRI group performance in the discrimination (d prime) between A_1_xC_1_ and A_1_xC_2_ (Dependency violations, Dep) and between A_1_xC_1_ and C_1_xA_1_ (Positional violations, Cat).

Participants from both Behavioral and fMRI groups exhibited a similar pattern of results suggesting that they were able to significantly discriminate correct phrases from dependency violations only after the NADs block (Behavioral group NADs block: mean *d’* Dep = 1.14, std = 1.45, *t*(18) = 3.43, *p* < 0.01; Behavioral group Random block: mean *d’* Dep = 0.15, std = 0.54, *t*(18) = 1.17, *p* > 0.25; fMRI group NADs block: mean *d’* Dep = 0.36, std = 0.96, *t*(30) = 2.1, *p* < 0.04; fMRI group Random block: mean *d’* Dep = 0.12, std = 0.49, *t*(30) = 1.3, *p* > 0.19). Both groups could discriminate correct phrases from positional (i.e. order) violations after both NADs and Random blocks (Behavioral group NADs block: mean *d’* Cat = 1.93, std = 1.02, *t*(18) = 8.27, *p* < 0.001; Behavioral group Random block: mean *d’* Cat = 1.84, std = 1.17, *t*(18) = 6.83, *p* < 0.001; fMRI group NADs block: mean *d’* Cat = 1.36, std = 1.12, *t*(30) = 6.75, *p* < 0.001; fMRI group Random block: mean *d’* Cat = 1.37, std = 1.01, *t*(30) = 7.55, *p* < 0.001) (see S6 Fig). Data used to generate S1 Text can be found in S4 Data.
