# Supplementary material for: Language statistical learning responds to reinforcement learning principles rooted in the striatum
Source: PLoS Biol. 2021 Sep 7;19(9):e3001119. doi: 10.1371/journal.pbio.3001119 (PMC8448350; doi:10.1371/journal.pbio.3001119)
Supplement: Attachment — Submitted filename: Response_to_Reviewers.docx [file pbio.3001119.s016.docx]

REVIEWS:

**Reviewer #1**: This work applies a reinforcement learning model (a Temporal Difference model, TD) to account for behavioral and neural measures of Statistical Learning (SL) of non-adjacent dependencies. Data from two studies are reported. First is a behavioral experiment showing that the TD model predicts change in RTs over repetitions in a structured block of the SL task. The second is an fMRI study, including in-scanner behavioral data replicating the first experiment, plus showing that the model's parameter reflecting the extent of prediction associated with the first element within patterns is correlated with trial-level activation in the striatum. Together, the authors interpret these two pieces of data as support for a strong mechanistic role of prediction in SL processes.

My overall impression of the manuscript is positive. As someone who works on SL but has limited knowledge of reinforcement learning and its models, I found the manuscript to be novel and thought-provoking. The combination of computational modeling, behavioral data from online SL measures, and neuroimaging should also be applauded. At the same time, there are issues in this version of the manuscript that should be addressed. Below I start by reviewing conceptual issues, followed by methodological ones.

**We want to thank the reviewer for his/her positive comments about our work and for the thorough revision, which has helped us improve the quality of this manuscript greatly. Below, the reviewer will find point by point answers to his/her comments and concerns.**

1. Conceptual issues: The role of prediction in SL.

1.1 As mentioned above, two pieces of data support the link between prediction and SL: The TD model's ability to predict change in RT behaviorally and the correlation between the model's parameter reflecting prediction and activation in the striatum. Both points convincingly demonstrate involvement of prediction in the SL task, which is already an important contribution. What these findings do not show, however, is that prediction is the mechanism responsible for SL (which is what the authors argue throughout). Rather, both types of evidence are consistent with SL models where prediction is the consequence, not the driver. Specifically, the fact that a TD model performs better than chance (or better than a simple Rescorla-Wagner model that is not set to learn non-adjacent patterns) is consistent with theories where prediction is the consequence (more SL -> more predictions -> reduced RTs). Similarly, the fMRI evidence is also consistent with prior work showing striatal involvement in SL (which again can be a consequence of learning).  What is missing from the paper is a 'smoking gun' type of evidence, which ties SL performance directly to extent of prediction. For example, an analysis showing that a substantial part of the inter-individual variance in the SL task can be traced back to model's parameters/striatal involvement (rather than that there is striatal involvement, but that most of the variance is carried by activation in, say, the hippocampus- see next point). If such additional evidence is not available, the authors need to be more careful in both the framing and discussion/conclusions.

**We thank the reviewer for raising these important considerations, thus giving us the opportunity to add clarity to the manuscript in support of our claims. Note that sustaining that prediction may be causally implicated in SL is not to say that prediction is the mechanism responsible for SL. Rather, we see prediction as part of a domain general learning mechanism, embodied in the TD algorithm and rooted in striatal function, that allows for the extraction of regularities from the environment. In this algorithm, predictions are both cause and consequence; that is, there would be no prediction error without prediction, and no prediction updating without prediction error. In the case at hand, therefore, prediction learning is tantamount with SL – the observation of a behavior – insofar as what is being learned are statistical contingencies. And prediction updating may be understood as part of a mechanism that can yield that observed behavior. We have therefore clarified in the introduction that what we contend not that prediction is ‘the mechanism’ of SL but a driver of SL (page 3, lines 36-38):**

**“Interestingly, however, the status of prediction as the driver of SL, rather than a mere consequence of it, that is, its causal implication in learning, has not been explicitly investigated.”**

**The critical aspect, therefore, is that we are observing statistical learning as it unfolds over exposure, rather than looking at the final output post-exposure. We believe that spelling out this putative suite of computations linked to plausible neurobiological structures pushes SL research beyond the very common conflation in the field between SL (as an observation of the post-exposure learning of structured material) and its underlying mechanism (which may be a dedicated or, as we claim, a more general learning system). In order to make this point clear in our manuscript, we have rephrased a section of the introduction to emphasize the critical distinction between the learning dynamics and the output of the learning process (see page 3, lines 27-32):**

**“One of the main reasons for this important gap in the literature is that the vast majority of SL studies have focused on the output of learning, generally assessed via offline post-familiarization tests, rather than on the learning process itself (14,15). It is only recently that work on SL has started to shift towards the use of online measures of learning. Online measures afford a more detailed representation of the learning dynamics and thus offer the possibility of generating hypotheses about the computations underlying SL.”**

**Another possible reason for the reviewer’s concern is that our explanation of the TD model and how it is fit to each individual behavioral data may have been insufficiently clear. We have now extended this explanation. Insofar as each subject’s prediction estimates for each trial are introduced as a parametric regressor for their own fMRI data (that is, at the first level), one can understand the reported striatal activity to reflect prediction learning that considers inter-individual variability both behaviorally and neuro-physiologically, which is what the reviewer was requesting as definitive evidence. In addition, the manuscript now also includes an additional figure in *Materials and methods* (Fig 6) that further details a trial’s computations for the model. We refer the reviewer to the ‘Temporal Difference model’ section in *Materials and methods* (pages 21-24, lines 510-590) of the manuscript, which has been substantially reworked. We apologize for the lack of clarity in the previous version of the manuscript. We hope these changes make it clear that the TD model is not fit to the entire behavioral dataset at once, but rather to each individual subject separately, accounting for inter-individual variability. Additionally, we now more clearly emphasize how prediction values for each trial are introduced as a regressor for each subject’s fMRI data at the first level of analysis. For this, we have included a new supplementary figure (S5 Fig) that shows the first level fMRI model fit to each subject’s data and the first level contrasts we performed.**

**Finally, we note that reports of striatal activation in classic SL tasks have mostly remained descriptive (e.g., Turk-Browne et al., 2009; Karuza et al., 2013) and rarely make claims about its specific contribution (with some notable exceptions, e.g., Giorgio et al., 2018; Karlaftis et al., 2018). We thus agree with the reviewer in that striatal involvement in our task is consistent with prior reports. However, we point out that our contribution is precisely to relate this involvement (at the individual level) to a very specific computation (prediction updating/error) that adheres to the learning trajectories of individuals and so captures the individual differences in this process. Having said this, we do not discard the existence of alternative learning systems capable of SL. We again thank the reviewer for raising this latter point, and thus the opportunity for us to discuss these alternative models further in our manuscript (see points 1.2 and 1.3 below).**

1.2 A more detailed account of how the current theory/model differs from other models of SL is needed.  Specifically, the authors should consider recent models that tie SL to the hippocampus (Schapiro et al., 2017 is a good example for a neurobiologically plausible computational work), as well as to network-level representation of uncertainty (Hasson, 2017). Is the current model meant to replace these models or complement them? What does the current model provide that these models do not? What type of evidence is needed to adjudicate between the current model and these previous attempts? In general, and in the same line of thought as the point above, any direct evidence of the current model's added value compared to existing models would further increase the impact of this paper.

**We thank the reviewer for pointing out the need for a broader view of our findings and interpretation in the context of other models of SL. There are several important differences between Schapiro and colleagues’ work and ours worth noting and that speak to the complementary nature our research. First, although Schapiro et al.’s model is applied to sequences of stimuli, they capitalize on the relationship between *pairs* of stimuli. Because of this, it is unclear how well such a model, which does not take non-adjacent relations explicitly into account, would fare in a task like ours where the relationship between adjacent elements is always extremely weak. In fact, the TD model ‘learns’ thanks to the low TPs between the consecutive (AX and XC) elements (cf. Gomez, 2002) and the temporal discounting parameter, which explicitly deals with the distance between predictor and predicted. Thus, striatal involvement could potentially be boosted by the nature of this particular SL task, and it is possible that alternative/concurrent learning mechanisms may operate when stimuli to be associated are contiguous rather than non-adjacent. Indeed, a direct comparison between materials containing adjacent versus non-adjacent relations would be a good test to contrast the different models and would likely help to better determine the implication and extend of hippocampal and striatal computations. From this point of view, hippocampal and striatal computations would work in parallel while more suited for the detection of adjacent versus non-adjacent dependencies, respectively. Indeed, recent data indicates that the MTL/hippocampus are sensitive to the identity of the elements that co-occur in an adjacent sequence (Henin et al., 2021; Karlaftis et al., 2018). The striatum in contrast appears to be engaged in probabilistic learning (Knowlton et al, 1996; Frank et al., 2004) and may therefore be more necessary in situations where uncertainty is present (Hasson, 2017). From this point of view, the basal ganglia would perform the trial-by-trial probabilistic computations and as a result of these computations eventually chunks of input could be extracted. The hippocampal/MTL declarative system may come into play when these episodic memories of the units in the input have been extracted (Opitz 2003, 2004; Henin et al., 2021). Therefore, in contrast to trial-by-trial sensitivity of the basal ganglia, hippocampal/MTL engagement under this light should correlate with aggregate measures of SL (see S5 Fig) rather than with *online* SL.**

**We performed a set of additional analyses to assess this possibility. Specifically, we covaried the results of the contrast between NADs and Random blocks with the overall difference in the mean RTs of each of the two blocks as an aggregate measure of SL. This analysis does show residual left anterior parahippocampal activity, along with widespread activity in the left insula, amygdala, posterior STG, and supramarginal gyrus (see Figure 1 below; Fig 5 in the manuscript). We have now included this contrast as Fig 5 in the manuscript and reported on it appropriately in the *Results* section (pages 11-12, lines 254 – 262). In contrast, in fMRI contrasts assessing brain activity covarying with trial-by-trial measures of NAD learning (*p*(A))(vs. baseline or vs Random *p*(X1)), or in the more general NADs vs. Random contrast, we found no trace of hippocampal involvement even when applying hippocampal masks (right and left separately) or small volume corrections.**

**We comment on these different points in the discussion, now broadly extended to take into account alternative learning systems capable of SL (pages 15-16, lines 365 - 393).**

**Figure 1 (Fig 5 in the manuscript). Brain regions related to mean statistical learning (NADs block vs. Random block activity covarying with the NADs effect).** Significant brain activity for the NADs block minus the Random block covarying with the NADs effect, i.e., an aggregate measure of SL. Results are reported for clusters FWE-corrected at p < 0.001 at the cluster level (minimum cluster size = 20). MNI coordinates were used. BA, Brodmann Area.

1.3 Relatedly, I'd appreciate a discussion of the relation between the current work and models that take part in SL phenomena within other domains. For example, in the context of reading, connectionist models that use prediction errors to learn the input structure are widely used (starting from Seidenberg & McClelland, 1989), and these models are now increasingly viewed as participating in SL of the written input (see Sawi & Rueckl, 2019; Seidenberg, 2011). Are there ways in which the current model is conceptually different from such connectionist models, which are used to learn statistical regularities outside 'typical' SL tasks?

**This is certainly a very interesting line of research with undoubtable similarities with our proposal. Neural networks of the kind described in these works implement error-driven learning (network weight updates) through algorithms such as error back-propagation and gradient descent for optimization. Although the neurobiological reality of these algorithms is still hotly debated, there is admittedly an element of similarity in the way prediction error is used for learning.**

**Nevertheless, as Sawi and Rueckl (2019) point out, a fundamental difference appears to be the type of representations they capitalize on. Applied to our specific case of SL, it is possible, that the BG system makes use of motor-articulatory representations, in contrast to the MTL system as it relates to connectionist networks, whose learning may be hypothesized to leverage sensory (e.g., auditory, visual) representations (if claims about representational formats are made at all). This is mentioned in the Discussion section in relation to the implication of the putamen in our task. Specifically, in the previous version of the manuscript, we speculated that ‘putaminal activity may reflect(s) the selection of covert motor responses, i.e., of (speech) motor programs corresponding to the predicted (C) elements […] (which, in turn) may be used to generate sensory-level predictions...’ This section of the *Discussion* has now been reworked in this review of the manuscript to make the point clearer (page 15, lines 343-352):**

**“The appropriate behavioral alternative (in our case, pertaining to the identity of the final C element of each phrase) may be finally concretized as a specific motor-articulatory representation of the element selected by the putamen from speech pre/motor areas used to predict upcoming auditory input (41). This selection of a ‘covert’ motor response is consistent with the attenuated (though not eliminated) activity in the putamen when regressing out overt motor responses (button presses; S3 Fig; cf. Fig 4). This cascade of processes, with the final selection of motor-articulatory representations by the putamen, may be used to generate the corresponding auditory predictions(41), ultimately translating into increasingly faster RTs for the predicted C elements. In this view, activity in the pSTG (Fig 4 and Fig 5) would reflect the downstream (i.e. sensory) consequences of this selection(41,71).”**

**We also speculate (page 15, lines 360-364) that “this prediction mechanism via motor-articulatory representations for learning should become of critical importance when putative alternative learning mechanisms (e.g., hippocampus; see below) are weakest, for example, when a temporal separation is imposed between the elements to be associated, as in our NAD learning task.”**

**As in the reading literature, these conjectures are consistent with the observation of different developmental time-courses for different forms of SL (e.g., adjacent vs non-adjacent), perhaps paralleling the development of the different learning systems (i.e., MTL and BG). It is also in line with the implication of the BG predominantly in SL tasks that that involve the association between non-adjacent elements (e.g., Karuza et al., 2013; Newport and Aslin, 2004), including our own, and the hypothesis that languages in which morphological regularities involve non-adjacent phonemes/letters (such as Hebrew or Arabic) may rely more heavily on the BG system (cf. Newport and Aslin, 2004). We now further emphasize in the Discussion section how a prediction-based learning mechanism rooted in striatal function differs from putative alternative learning systems and under what circumstances we believe the BG learning system might be more necessary (see pages 15-16, lines 365-393).**

2. Methodological issues: Clarification of computational and statistical models.

2.1 The description of the methods is generally clear, except for the description of how RTs in the task were used to derive the model's parameters. On p. 18, it is stated that "[t]o obtain the free parameters for each participant … [w]e selected the values that produced the minimum Log-Likelihood Estimate (LLE), indicating the best possible fit between the model predictions and the participant's transformed RTs." This is unclear. What was the function that linked the model's parameters to the RT data?

**We thank the reviewer for raising this important point. As also noted by other reviewers, the description of the model was insufficiently clear and potentially confusing. As pointed out above, we have now clarified and extended this description greatly throughout the Temporal Difference model section in *Materials and methods* (pages 21-24, lines 510-590). Within this description, we have also further detailed how the RTs were used to derive the model parameters. In particular, we specify that:**

**‘As a behavioral index of participants’ predictive capacities, we used reaction times, since RTs should be faster when the associations are learnt than when they are not. Reaction times were first standardized (z scored; zRT) and then normalized between 0 and 1 by the softmax type function:**

**xRT = (1./(1 + exp(zRTs))).**

**Note that this function will output larger xRT values for lower input zRT values (and conversely, smaller xRTs values for higher input zRTs), in accord with the idea that better predictive capacities will elicit faster RTs. Importantly, the function also minimizes the effect of extreme RT values.**

**To fit the free model parameters to each participant’s responses, we assumed the following function to minimize the difference between the absolute value of V(A^i^) and the transformed reaction time in a given trial t:**

**Δ_t_ = 1 – abs(xRT^i^_t_ – abs(*V*(A^i^)_t_ ))	*(equation 6)***

**We then selected the α and γ values that produced the maximum Log-Likelihood Estimate (LLE), indicating the best possible fit between the model predictions and the participant’s transformed RTs.’**

2.2 The statistical approach used in the fMRI analysis is underspecified (and potentially flawed). It seems that the authors used a 'simple' GLM to examine the relation between trial-level estimated parameters (from the model) and the trial-level activation (after controlling for additional factors). But, trials are nested within subjects (i.e., multiple trials with different values within subjects), and thus a simple GLM may not be appropriate. If this is indeed the case, please correct and use an a statistical method that deals with the nesting of trials within subjects (e.g., mixed effect models; see Chen et al. 2013). If my reading of the text is incorrect, please clarify.

**We apologize for the lack of clarity in describing our fMRI analysis. We have introduced a new supplementary figure (S5 Fig) that specifies the first-level fMRI model used along with the contrasts performed for each subject before deriving group level statistics. We hope that this clarifies our individual differences approach and helps readers understand the reported relationship between trial-wise SL performance and brain activity.**

**Reviewer #2**: This paper asks about the dynamic of statistical learning and its neural bases presenting first a behavioral experiment and then an fMRI one of the same task. Participants were asked to respond as quickly as possible to target pseudo-words. The predictability of these targets was manipulated within subjects between two task versions. While the three pseudo-words of each trial phrase were random in a control version, in the learning version the first pseudo-word was perfectly predictive of the last pseudo-word of each phrase. In that predictive version, the participants' response times to the final word elements decreased as they learned to anticipate the final element from the predictive first element. A reinforcement learning algorithm was fit to participants' data, with the resulting fits being used to analyze behavioral data and extract learning parameters, which were then related to BOLD signal. Primary analyses probed the relations between trials' BOLD signal and reinforcement learning algorithm fits. The authors conclude that a Temporal Difference model provides a good algorithmic description of learning during the predictive task. The authors then further present an algorithmic interpretation of neural correlates of learning including striatal activity.

While providing a rich and interesting read, this paper suffers from two major weaknesses that should be addressed before it can be considered fit for publication:

**1.**The authors state in the abstract and introduction that their focus is on revealing the neural bases of statistical learning as it unfolds but their paper suffers throughout from a rather fuzzy use of the terms "statistical learning,"  "rule learning", and "reinforcement learning." Mechanistically, the literature points to these various forms of learning as having distinct characteristics. Yet, it remains unclear to this reader whether this paper focuses on statistical learning or another form of learning. Indeed, the actual predictive task used by the authors is best described (as the authors do in their Methods) as "rule learning.", and not statistical learning.  Participants simply have to learn a one-to-one, deterministic correspondence between a cue and a response. The learning challenge is made less trivial by the insertion of a long-distance dependency - yet it seems best characterized as an instance of associative learning, in this case entirely deterministic and quite unlike what is labeled statistical learning in the literature referenced by the authors. To the extent that a purely deterministic system's contingencies do not have statistical variation, the authors' claims about many putative processes, such as prediction error, appear to be at odds with the actual task completed by participants. While the authors claim that the close correspondence between the TD fits and the RT are evidence for particular mechanisms, it seems likely that the TD model is simply very flexible and is able to take the shape of the data. For example, it is not clear why change in RT would be nonmonotonic, as shown in Figures 1 and 2 (i.e., sometimes increasing with learning). The TD model would predict that RT should increase when expectations are violated. But, in a deterministic system with fairly simple rules, the participants' expectations were never violated by the stimuli. As the core novelty of this paper is based on applying reinforcement
learning algorithm to a task situation of statistical learning, a task that consists of learning deterministic associations would be entirely at odd with the paper stated goal. How the main learning task consists into a form of statistical learning should be clarified.

**We thank the reviewer for the very insightful thoughts on these important issues. We fully understand the reviewer’s concerns and apologize for the lack of clarity. We review here potential sources of confusion in this point-by-point answer and detail for each of them how it was addressed in the review of the manuscript.**

a) **One potential source of confusion, as pointed out by the reviewer, is the concept of ‘rule learning’. This term was used in related literature using similar non-adjacent dependencies and we adopted it for the sake of facilitating direct comparison. However, we fully agree this can be misleading, since ‘rule learning’ implies a level of abstraction that is not the focus of the present paper. It is certain that our behavioral task can and has been used to study abstract rule learning (e.g., López-Barroso et al., 2016; Orpella et al., 2020). In these previous works, we tested rule learning by probing participants’ ability to generalize the learned syntactic structure to a new artificial language and/or to new strings following the same structure. Importantly, in Orpella et al. (2020), we show that SL differs from rule abstraction, which occurs at a later stage and capitalizes on a different set of processes and brain structures. In any case, as the reviewer points out, the focus of the current study is on the early stages of learning when distributional information is learned from the input, not on the later stages of rule abstraction. Accordingly, we have changed all references to rule learning to *non-adjacent dependency* learning throughout the manuscript. In addition, we have better and more explicitly specified the object of study of the current paper as distinct from abstract rule learning (page 4, lines 60-68):**

**“In natural languages, non-adjacent dependencies (henceforth, NADs) are abundant and underly important morphological and syntactic probabilistic rules (e.g., the relationship between *un* and *able* in *un*believ*able*). Sensitivity to NADs is therefore important in the early stages of grammar learning, when the relation between phrase elements is tracked at a superficial level and before more abstract representations (syntactic rules) can be created via other mechanisms and brain structures(33). However, sensitivity to NADs can also be critical for speech segmentation in the early stages of word learning(34), both in prelexical-development(2) and beyond (i.e., second language acquisition(35)).”**

b) **The other term that may have needed justification is “statistical learning”. A wide range of tasks have been used under the concept of statistical learning and this has been an issue that calls for a specification of the different dimensions comprised within this umbrella term (e.g., the type of regularity, such as spatial or sequential regularities, adjacent vs non-adjacent dependencies; or the input modality), as highlighted by several reviews on the topic (Sawi and Rueckl, 2019; Hasson, 2017). The aim of our study is to contribute to the understanding of the computations underlying statistical learning of non-adjacent dependencies, which are a specific but critical type of regularity in most languages. Critically, however, despite being specific, this type of SL seems to rely on the same domain-general mechanisms used in the learning of other types of statistical/probabilistic relations outside the linguistic domain. The justification for using the term SL for this task further comes from the use of the same type of non-adjacent dependency material in the classic statistical learning literature (e.g., Misyak, Christiansen and Tomblin, 2010), including works by Saffran and colleagues (e.g., Saffran and Wilson, 2003; Newport and Aslin, 2004). Here, SL is broadly defined as the ability to extract patterns or regularities in the environment (Aslin and Newport, 2012; Shapiro and Turk-Brown, 2015). This definition is introduced in the first sentence of the abstract and the paper to set the frame of our study.**

c) **The reviewer also tightly relates this criticism on the use of the term SL to the fact that there is a deterministic relation between the non-adjacent dependencies, which s/he claims to be misaligned with the use of a reinforcement learning algorithm to model this type of learning. There are two aspects that need to be clarified in relation to this point. On the one hand, we realized also from point 1.1 of Reviewer #1, that the way the model was implemented was not sufficiently spelled out. Although a trial in the experimental task corresponds to a three-word phrase, the model is fed each word independently (A, X, C) and generates predictions and prediction errors at each element of the continuous sequence. We talk about trial-by-trial predictions for the model because the focus is on changes in parameter A, which corresponds to the first element of each phrase and the beginning of each experimental trial. Therefore, although the non-adjacent relations are deterministic, the adjacent relations, which are fed into the model, are not. In fact, most X elements could equally occur after each of the two As. These variations in probabilistic information between adjacent and non-adjacent relations are the core interest of the material used and render a TD model more appropriate in comparison with other models (e.g., Schapiro et al, 2016). These other models capitalize on the relationship between *pairs* of adjacent stimuli and have been used to model SL tasks that have equally deterministic relations between, albeit between adjacent elements (see response to point 1.2, Reviewer #1). On the other hand, we also believe that it is legitimate to speak of prediction error. By definition, a prediction error may occur in any circumstance where a prediction is not met. Note that predictions need not be necessarily categorical (e.g., a prediction of specific element X vs element Y) but can involve graded predictions (i.e., predictive *values* regarding element X or Y), as implied by the TD model. As such, a prediction error will follow the presentation of any element that is not *fully* predicted (e.g., prediction value < 1). In line with this, in this work we hypothesized and aim to show that both predictions and prediction errors appear to be important elements of the SL mechanism or, more precisely, of the learning mechanism that affords SL. We respectfully disagree with the reviewer in that ‘participants' expectations were never violated by the stimuli’. Their expectations we in fact violated at every step of each experiment’s trial, relative to the predictive value carried by each element of the phrase. Finally, in relation to the nonmonotonic RTs decreases mentioned by the reviewer, these are expected given the pseudorandom presentation of two different deterministic structures (*target* and *no target*).**

**In accord with these important considerations, we have now extended the description of the model’s computations in the Temporal Difference model section in Materials and methods (pages 21-24, lines 510-590) and added a new figure (Fig 6) in *Materials and methods* to explain how prediction errors are generated at every step and can be used even in a deterministic system. We have also made important changes in the nomenclature to better conform with the RL literature in our explanation of the TD model, which should also make the graded nature of predictions in this context clearer. The key is in understanding that elements carry a predictive *value* in respect to upcoming elements, and that this is what increases over time (i.e., this is the learning itself).**

**2.**The authors' framing of their field, and their results in that field, repeatedly refer to the potential for bidirectional causality between changes in predictions and changes in statistical learning. This point would benefit from being carefully argued for. The authors never explain why statistical learning could not be instantiated by learning to predict; that is, the change in prediction *is* the learning or in other words, diagnostic of the behavior of a system demonstrating the presence of learning. The authors describe their trial-by-trial estimates of predictions, and clearly they have trial-by-trial BOLD measurements. Given the focus of the authors on the causal directionality of prediction vs. learning, it seems analyses examining the temporal order of the development of BOLD measurements in relation to the development of the prediction estimates should be in order at a finer-grained timescale than aggregating across their entire blocks as they do. Using a more precise timescale of analysis may help the authors in other ways as well. For example, the results in line 140 are surprisingly weak, which may have been because the authors averaged across the entire behavioral blocks. If the intention was to test for learning, rather than performing analysis by block, the authors could show that RTs decrease at a steeper rate and to a greater extent in the predictive than random block. This particular problem may also be addressed in part by using mixed-effects models with by-participant random effects rather than averaging within participants and then using ANOVA.

**We thank the reviewer for this suggestion that has contributed to making our results and aims clearer. In line with the reviewer’s suggestion, we have now performed linear mixed models analyses for both groups (behavioral only and fMRI) and detailed the results in the manuscript (Fig 2A and Fig 3A) and text lines 115 to 123 and lines 176 to 178. We agree in that a direct comparison between the slopes for the structured condition (NADs block) and the unstructured condition (Random block) further the previously reported behavioral results and contribute to understanding the gradual nature of learning over trials. However, we have kept the analysis of variance for its intuitive appeal and link to previous behavioral research (López-Barroso et al., 2016) but particularly since the so-called NADs effect (the difference in mean RT between the NAD block and the Random block) is later employed (Fig 5) to emphasize the difference between correlates of aggregated data (i.e., block means) and online measures of SL.**

**Further, we apologize for the lack of clarity regarding our fMRI analysis. We note that this analysis does exactly what the reviewer suggests, i.e., correlates the trial-by-trial measure of prediction for each participant with their own trial-by-trial BOLD signal measurement. To make this clearer in the manuscript, we have now extended the explanation of the fMRI models employed and included a new figure in the SI (S5 Fig) detailing all the regressors used. Lines 619 to 638 (pages 25-26) in the fMRI preprocessing and analysis section of *Materials and methods* now read:**

**“To identify brain regions related to the trial-by-trial development of subjects’ predictions/prediction errors, a model with the conditions NADs Target, NADs No Target, Random Target and Random No Target, and all offline test conditions (see Supporting Information) was specified at the first level. This also included, in first place and for each trial for each of the conditions of interest (NADs Target, NADs No Target, Random Target and Random No Target), a parametric modulator (a vector) corresponding to the RT (z-scored and inverted); and in second, a parametric modulator (a vector) corresponding to the trial-by-trial prediction/prediction error (*p*(A), also z-scored; (S5 Fig)). […] We additionally included, in all the models described above, the movement parameter estimates for each subject computed during preprocessing to minimize the impact of head movement on the data (S5 Fig).**

**For each participant, the following contrasts were calculated at the first level (S5 Fig):**

- **NADs z*P*(A) vs implicit baseline**
- **NADs z*P*(A) vs. Random z*P*(X1)**
- **NADs z*P*(A) vs NADs zinvRT**
- **NADs z*P*(A) vs Random zinvRT**

**These were subsequently entered into corresponding one-sample *t*-tests *at the second level* to arrive at the reported fMRI results.”**

Apart from these two main comments, we have a number of other questions about the work:

**3**. Another source of worry from this reader concerns the definitions of clusters using a whole-brain searchlight using the test statistics from p(A) as a predictor within a mixed-effects model. The minimum cluster size and related details are mentioned line 491, but beyond this brief text, it's not really clear how the earlier explanations (e.g., discussing controlling for motor response) relate to the actual cluster definitions. In particular, when lines 167-168 say "we correlated this proxy for prediction learning with participants' trial-wise BOLD signal measures for the Rule block" it is difficult for the reader to know what is actually happening in terms of model fitting/assessment. While the Result section is indeed not the place to thoroughly explain such a procedure, the authors could ensure that an interested reader can determine exactly how this "correlation" was implemented.

**We thank the reviewer for the useful comment. As mentioned in point 2, we have now clarified the fMRI models in the form of a figure in the *Supporting Information* (S5 Fig) and an extended explanation in the *Materials and methods* section. Here the reader can now more clearly see the details of the fMRI model fit to each participant’s data and the contrasts performed at the first-level. We have also specified the threshold for statistical significance for the fMRI one-sample contrasts in the results section of the manuscript (lines 205-206, page 9). Furthermore, we have rerun all fMRI contrasts under the same first-level model for simplicity’s sake and to increase the homogeneity across the fMRI analyses. We note that, because of this, tables and figures may have changed slightly, but that this modification has absolutely no impact on any of the results (beyond increasing the significance of most clusters), and therefore on their interpretation.**

**4**.The authors mention that TD models may be particularly able to capture the computational features of learning associated subcortical activity (lines 303-304 - "a possible explanation for this concerns the emphasis that particular models of learning place on specific computations, with TD models being most sensitive to subcortical (rather than neo-cortical) activity."). In fact, the authors have the ability to test this, don't they? It would seem appropriate to back up their statement with a model comparison between TD and other simpler models, such as RW in both subcortical and neocortical areas to properly document their point. If this particular contrast (i.e., between TD and other models' neural analogues) is not what the authors were referring to in line 304, then they should clarify.

**We apologize for the lack of clarity in this discussion point. This comment derives from what is mentioned in the cited paper of Karuza et al. (2014). In this paper, the authors speculate on how different models may be more able to bring out the activity of particular structures in relation to their computations. Specifically, in a section of their paper dedicated to computational modelling of SL they write: ‘Even models that have comparable validity might have parameters that are correlated with different neural regions (e.g., a R-W model might correlate well with regions of the striatum in a given task, while a Bayesian model might correlate with a region in the frontal cortex).’ To clarify then, by different models we were referring to models that in principle place emphasis on different type of computations. The RW model uses similar type of computations as the TD model, the main difference being the inclusion of a decay parameter in the TD model to account for the (temporal) distance between the prediction and the predicted. Because of this, and because of its inferior fit to participants’ data, we do not think it would be informative to compare its neural correlates to those we report as related to the TD model. Note also that the search for the best fitting model was also not the object of our study. We agree with the reviewer in that we cannot make such a speculation from our data and the paragraph was potentially confusing, so we have removed that short paragraph from the *Discussion*. Instead, we have added a discussion of alternative models of SL in the literature, including neural network models of the hippocampus (e.g., Schapiro et al., 2016; lines 365 to 393, pages 15-16) which we believe enrich the relevance of our proposal.**

**5**. A couple points regarding model fitting:
- Why were z-scored RTs exponentiated prior to normalizing between 0 and 1 (line 419)? Does this pertain to the specific distributional assumptions of the TD model? RT distributions are typically quite skewed, and the authors note that there are "fluctuations due to decision processes, motor response preparation and execution, random waxing and waning of attention, and system noise". This is true, and these are reasons that rare large RTs have disproportionate effect on, say, mean RT values. In fact, RTs are often log-transformed in order to better approximate normality (although this procedure has its own problems), but exponentiating the distribution of RTs would have the opposite effect, and only *increase the influence* of the rare and large RT data points.

**We thank the reviewer for pointing out the lack of clarity of our normalization procedure. We normalized participant’s RTs according to a standard softmax function. This is shown in Figure 2 below. This function is often used in the domain of RL to convert values into action probabilities and, as can be appreciated in the figure, it will also minimize the impact of extreme values (RTs in our case), rather than increase their influence. We additionally limited participants’ responses to 1 second, as detailed in the manuscript’s *Materials and methods* section lines 480-481, which also minimized the presence of extreme RT values caused by distraction, fatigue, etc. We have extended the explanation of how each participant’s RT were treated and used to fit the TD model’s free parameters as follows (lines 554-565, page 23):**

**“Reaction times were first standardized (z scored; zRT) and then normalized between 0 and 1 by the softmax type function:**

**xRT = (1./(1 + exp(zRTs))).**

**Note that this function will output larger xRT values for lower input zRT values (and conversely, smaller xRTs values for higher input zRTs), in accord with the idea that better predictive capacities will elicit faster RTs. Importantly, the function also minimizes the effect of extreme RT values.**

**To fit the free model parameters to each participant’s responses, we assumed the following function to minimize the difference between the absolute value of V(A^i^) and the transformed reaction time in a given trial t:**

**Δ_t_ = 1 – abs(xRT^i^_t_ – abs(*V*(A^i^)_t_ ))	(equation 6)”**

**Figure 2.** Softmax function used to bind zRTs between 0 and 1 and limit the effect of extreme RT values.

- On line 420, did the authors meat that their parameter optimization found the maximum log-likelihood estimate? The minimum log-likelihood is probably not defined for their function (if their optimization problem is typically concave), nor would it be theoretically interesting. A common approach to maximum-likelihood estimation is to minimize the negative log-likelihood; it's possible the authors reversed this in their explanation.

**We apologize for the mistake and thank the reviewer for the catch. We have modified the explanation of the parameter optimization procedure to read (pages 23, line 566-568):**

**‘We then selected the *α* and *γ* values that produced the maximum Log-Likelihood Estimate (LLE), indicating the best possible fit between the model predictions and the participant’s transformed RTs.’**

**6.** Figure captions should clearly indicate the measure of variability. For example, in Figure 2, the caption describes "mean reaction times and error bars" and later in the caption it says that "vertical bars are the SD." However, I'm guessing that the initial statement about error bars does not refer to SD, but refers to either standard errors or confidence intervals.

**We have now replaced the bar graphs in (now) Fig 2 and Fig 3 in the manuscript by regression plots for the linear mixed model analyses. We thank the reviewer for pointing out the lack of clarity and hope these new analyses/plots convey the results more clearly.**

**7**. "Welcome Trust" (line 458) should be "Wellcome Trust"

**We thank the reviewer for catching this typo. We have now corrected it.**

**Reviewer #3**: Review for "Statistical learning as reinforcement learning phenomena" by Orpella and co. The authors aimed at investigating the computational and neural correlates of statistical learning in the context of artificial language acquisition. The run two experiments (one behavioural, the other fMRI) and found that statistical learning (as indexed by reaction times) is well explained by a temporal difference prediction error model (compared to a random model and a Rescorla-Wagner model) and that TD prediction errors are represented in the network encompassing the striatum and the ventral prefrontal cortex. The paper is very clear and the results seem robust. The topic is of interest for broad readership and I praise the authors for assessing the too-much-neglected role of reinforcement learning processes in language learning. I have several suggestions that I think could improve the paper. In each categories, the issues are ranked by order of importance.

**We thank the reviewer for the very positive appraisal of our work and for the many good suggestions that have no doubt helped to improve it greatly. Below, the reviewer will find a point-by-point response to his/her concerns and an explanation of how we have addressed them.**

-- behavioral task and results
1/ I strongly encourage the authors to include a figure with the task / stimuli. For people not working in language (as me) it is very hard to figure out what the subjects are actually seeing / listening to / doing. This should be the first figure of the paper and I also recommend to clearly specify to which time steps of the task the functional activations correspond to.

**We have now included a figure that details the behavioral paradigm. This is now Fig 1 in the manuscript and is depicted as follows:**

**Figure 3 (Fig 1 in the manuscript). NAD learning task.** The same procedure was used for the behavioral and fMRI groups, except for the intertrial interval (ITI), lasting 1 sec in the behavioral group and jittered between 1.5 and 3 secs in the fMRI group. Participants were requested to indicate, via a button press, whether the target word was present (YES) or absent (NO) after listening to each phrase, with a maximum of 1.5 sec allowed for response. Reaction times were calculated from the onset of the 3^rd^ word of each phrase. Trial (i.e., event) onsets for fMRI analyses were set to the beginning of each phrase.

**We apologize for the lack of clarity in the description of the time steps of the task corresponding to the functional activations. We have now clarified this description in the *Materials and methods* section (lines 462-470, page 19)*,* included a new figure with the details of the first level contrasts (Fig S5), and information in the legend of the figure describing the task (Fig 1 in the manuscript).**

2/ I strongly encourage the authors to report not only the mean ± sem (barplots), but also the individual points. It is becoming standard in the field and for good reasons.

**We have now complemented the initial behavioral analyses of participants’ SL performance with linear mixed effects models. Accordingly, we have changed the bar plots with plots of the (binned) raw data with SEM bars along with the predicted slopes from the linear mixed model (please see Fig 2 and Fig 3 in the manuscript for details).**

**Figure 4 (Fig 2 in the manuscript). Behavioral group’s SL and temporal difference (TD) model results. (A)** Slopes for NADs and Random blocks (N = 19) derived from the linear mixed model. A significantly steeper slope for the NADs block (red) compared to the Random block (black) indicates SL over trials. Actual data shown averaged into 6 trial bins with the SEM for display purposes only. All data points per participant were used for the linear mixed model analysis. **(B)** Plot of participants’ mean reaction times (blue) against the TD model’s estimates of the development of predictions over learning (red; inverted as 1-*P*(A) before averaging and z-scoring for display purposes). Vertical bars = SD. RTs = reaction times. Reaction times were initially transformed (see Materials and methods) and are plotted with the inverted model estimates in z-score values. *P*(A) = TD model’s predictions from the initial word (A) of the dependencies.

**Figure 5 (Fig 3 in the manuscript). fMRI group’s SL and TD model results.** Slopes for NADs and Random blocks (N = 31) derived from the linear mixed model. A significantly steeper slope for the NADs block (red) compared to the Random block (black) indicates SL over trials. Actual data shown averaged into 6 trial bins with the SEM for display purposes. All data points per participant were used for the linear mixed model analysis. **(B)** Plot of participants’ mean reaction times (blue) against the TD model’s estimates of the development of predictions over learning (red; inverted as 1-*P*(A) before averaging and z-scoring for display purposes). Vertical bars = SD. RTs = reaction times. Reaction times were initially transformed (Materials and methods) and are plotted with the inverted model estimates in z-score values. *P*(A) = TD model’s predictions from the initial word (A) of the dependencies.

-- fMRI

1/ My main issue concerning the fMRI results is the lack of clarity concerning the exact structure of the GLMs and the contrast used. I strongly encourage the authors to include a clear presentation of the GLMs and the contrast used, similar to this one:

<https://www.jneurosci.org/content/jneuro/suppl/2009/10/28/29.43.13465.DC1/Supplemental_Material.pdf>

**We thank the reviewer for the helpful suggestion. This has also helped us to clarify the concerns of Reviewer #1 (see responses to point 1.1 and 2.2) and Reviewer #2 (responses to points 2 and 3). We have now included an additional figure in the SI (S5 Fig) that specifies the model fit to each participant’s fMRI data and the contrasts performed at the first level (see Figure 6 below). We have also extended the** **fMRI preprocessing and analysis section in *Materials and methods* (lines 617 to 638, pages 25-26) to further specify the fMRI model and the contrasts performed.**

**Figure 6 (S5 Fig in the SI). fMRI first-level model and contrasts.** Model fit to each subject’s fMRI data and vectors used to compute the specified contrasts.

2/ Related to the previous issue, the manuscript lacks clarity concerning the preprocessing (z scoring or not?) of the parametric modulators and which parameters (individual or group level) have been used to generate the regressors. Please refer to Lebreton et al. Nature Human Behaviour (2019) for a full treatment of this issue in the context of model-based fMRI.

**We hope that the previously mentioned Fig S5 and the now extended explanation of the fMRI analysis in the reviewed manuscript makes also these details clear to the reader.**

3/ There are many interesting fMRI results (for example P(A) - Rule or P(A) - Norule) that are currently only presented in the SI. I believe they deserve a place in the supplementary materials. Also (see my previous point) I am not sure whether the authors report the basic reaction time contrast. Were the correlates of reaction times different across conditions?

**We did not include the fMRI contrasts reported in the SI in the main manuscript body to maintain the clarity and succinctness of the paper. We note that these contrasts were performed as control analyses to support the main points of the paper. We also note that some of these contrasts are in fact somewhat redundant. For example, given that SPM automatically orthogonalizes regressors and assigns any shared variance between regressors to the first regressor input in the model (in this case, the inverted and z-scored RTs), the betas for the *p*(A) regressor should reflect the correlation between measured brain activity and this parameter ‘controlling for’ the reaction time related activity of that block. Thus, the orthogonalized *p*(A) regressor measured the BOLD variance that is uniquely explained by this regressor, *over and above* what is explained by the RTs.**

**Following the reviewer’s suggestion, we also looked at differences across conditions in the RT contrasts. There were no significant clusters for the contrast between the reaction times of the two conditions (NADs vs Random) in any direction.**

4/ I also encourage the authors to check for the negative correlations of the prediction errors (there is an hypothesis about different neural systems processing PEs in opposite directions), for example in the insula or dorsomedial prefrontal cortex.

**We thank the reviewer for the suggestion. However, our fMRI contrast focuses on anticipatory processes (predictions) by looking at brain activations associated with the first element A of each phrase, rather than the P.E. itself as generated on elements X and C. Despite this, we have looked at brain activity that negatively correlated with individuals’ predictions to provide a response to the reviewer’s point. However, we did not find any significant results pertaining this particular contrast.**

5/ in addition to the activation tables, the authors should consider uploading the SPMs into [neurovault.org](http://neurovault.org/)

**We thank the reviewer for the suggestion. We have now uploaded our main contrasts to neurovault.org under the title of the article.**

6/ Concerning fMRI reporting, the authors should explicit the significance threshold used for the fMRI results in the Results sections ('lines 164 onward'). In the current version it is only specified in the methods section and in the legend.

**We thank the reviewer for catching this bit of missing information. This information has now been inserted in the main text as well in each of the relevant figures and *Materials and methods* section, following the reviewer’s recommendation. The main text, lines 205-206 (page 9) now reads:**

**‘We report results for clusters FWE-corrected at p < 0.001 at the cluster level (minimum cluster size = 20).’**

-- computational modeling

1/ The notation used by the authors is really not-standard for the TD framework. I actually had trouble convincing myself that the model was roughly equivalent to what I know as the TD-learning from Sutton and Barto's book. The authors should consider rewriting the formalism in a more standard way.

**We apologize for changes in the standard notation of the TD model. Originally, we made these changes to better integrate the RL and the language learning domains. For example, by referring to state values as predictions, we aimed to more intuitively convey what state values afford. In line with the reviewer’s suggestion, however, we have now changed the model’s description in the methods section to conform to more standard formalisms in the field (lines 510 to 590, pages 21-24). For instance, state values are now termed *V*(A^i^)t and *V*(X^i^)t, and a trial’s outcome is now termed *R* as it is common in the RL literature. Nevertheless, we have maintained the terms previously used in the main text (e.g., *p*(A) instead of *V*(A)) to make the paper’s focus more accessible to the broader readership, and explained the correspondence appropriately in the *Materials and methods* section lines 548 to 552 (pages 22-23), which read:**

**“The absolute value of *V*(A^i^) reflects its predictive capacities and the associative strength between element A and a particular response, with higher values indicating stronger predictions. Because of this, we have replaced the formal term *V*(A) by the alternative term *p*(A) throughout the manuscript, as *p*(A) may be more intuitively related to *predictions from element A* by the general reader than *V*(A).”**

2/ The authors should also explicit the equations of the RW model. I assumed it's equivalent to the TD, but with Gamma=0? A non-expert will need this information.

**We apologize for the lack of clarity regarding the equations underlying the RW model. Indeed, the main difference with the TD is that the RW model has no Gamma parameter. We also summed the predictions from the A and X element to form a total prediction for each trial as suggested in Niv and Montague (2009). These details are now specified in a new section under *Materials and methods* titled *Rescorla-Wagner model*, page 24, lines 591-597, which reads:**

**“The Rescorla-Wagner model was specified as follows: in each trial the state values *V*(A^i^)_t_ and *V*(X^i^)_t_ were summed to produce a single prediction *V*(A^i^X^i^)_t_ per trial. No P.E. was computed on the 2^nd^ element of each phrase (e.g., X in NAD phrases). A P.E. was computed for each C element as *∂* = *R* – *V*(A^i^X^i^)_t_. This was then used to update both *V*(A^i^)t and *V*(X^i^)_t_ as *V*(A^i^)_t+1_ = *V*(A^i^)_t_ + *α · ∂* and *V*(X^i^) _t+1_ = *V*(X^i^)_t_ + *α · ∂*, respectively. Note that this is equivalent to the TD model’s updates without the *γ* term.”**

-- discussion
1/ The authors should consider citing / discussing Giavazzi et al Cortex (2018), which is, I believe, consistent with their caudate results.

**We want to thank the reviewer for pointing our attention to this very interesting work by Giavazzi et al. that clearly converges with our results and helps us in the interpretation of the role of the caudate in this framework. We have now included a mention of their results in relation to our work and added discussion on this interpretation integrating it with our work.** **This reads (page 14, lines 333-336):**

**“In agreement with our results, recent data (64) suggests that this role also applies to the language domain, with evidence for an implication of the caudate nucleus in the selection of linguistic alternatives from left prefrontal perisylvian regions with which it is connected(65).”**

-- typos

1/ Line 248 "oft-reported"

**We have changed it to “oft-cited”.**

**References**

Turk-Browne NB, Scholl BJ, Chun MM, Johnson MK. Neural evidence of statistical learning: efficient detection of visual regularities without awareness. J Cogn Neurosci. 2009 Oct;21(10):1934-45. doi: 10.1162/jocn.2009.21131. PMID: 18823241; PMCID: PMC2773825.

Karuza, E. A., Newport, E. L., Aslin, R. N., Starling, S. J., Tivarus, M. E., & Bavelier, D. (2013). The neural correlates of statistical learning in a word segmentation task: An fMRI study. *Brain and language*, *127*(1), 46–54. https://doi.org/10.1016/j.bandl.2012.11.007

Joseph Giorgio, Vasilis M. Karlaftis, Rui Wang, Yuan Shen, Peter Tino, Andrew Welchman, Zoe Kourtzi, Functional brain networks for learning predictive statistics, Cortex, Volume 107, 2018, Pages 204-219, ISSN 0010-9452, https://doi.org/10.1016/j.cortex.2017.08.014.

Vasilis M. Karlaftis, Rui Wang, Yuan Shen, Peter Tino, Guy Williams, Andrew E. Welchman, Zoe Kourtzi

White-Matter Pathways for Statistical Learning of Temporal Structures. eNeuro 29 June 2018, 5 (3) ENEURO.0382-17.2018; DOI: 10.1523/ENEURO.0382-17.2018

Gómez RL. Variability and Detection of Invariant Structure. *Psychological Science*. 2002;13(5):431-436. doi:10.1111/1467-9280.00476

Henin S, Turk-Browne NB, Friedman D, Liu A, Dugan P, Flinker A, Doyle W, Devinsky O, Melloni L. Learning hierarchical sequence representations across human cortex and hippocampus. Sci Adv. 2021 Feb 19;7(8): doi: 10.1126/sciadv.abc4530. PMID: 33608265; PMCID: PMC7895424.

Knowlton BJ, Mangels JA, Squire LR. A neostriatal habit learning system in humans. Science. 1996 Sep 6;273(5280):1399-402. doi: 10.1126/science.273.5280.1399. PMID: 8703077

Frank MJ, Seeberger LC, O'reilly RC. By carrot or by stick: cognitive reinforcement learning in parkinsonism. Science. 2004 Dec 10;306(5703):1940-3. doi: 10.1126/science.1102941. Epub 2004 Nov 4. PMID: 15528409.

Hasson Uri 2017 The neurobiology of uncertainty: implications for statistical learningPhil. Trans. R. Soc.

http://doi.org/10.1098/rstb.2016.0048

Opitz B, Friederici AD. Interactions of the hippocampal system and the prefrontal cortex in learning language-like rules. Neuroimage. 2003 Aug;19(4):1730-7. doi: 10.1016/s1053-8119(03)00170-8. PMID: 12948727.

Opitz B, Friederici AD. Brain correlates of language learning: the neuronal dissociation of rule-based versus similarity-based learning. J Neurosci. 2004 Sep 29;24(39):8436-40. doi: 10.1523/JNEUROSCI.2220-04.2004. PMID: 15456816; PMCID: PMC6729909.

Newport EL, Aslin RN. Learning at a distance I. Statistical learning of non-adjacent dependencies. Cogn Psychol. 2004 Mar;48(2):127-62. doi: 10.1016/s0010-0285(03)00128-2. PMID: 14732409.

Diana López-Barroso, David Cucurell, Antoni Rodríguez-Fornells, Ruth de Diego-Balaguer, Attentional effects on rule extraction and consolidation from speech,Cognition, Volume 152, 2016, Pages 61-69, ISSN 0010-0277, https://doi.org/10.1016/j.cognition.2016.03.016.

Orpella J, Ripollés P, Ruzzoli M, Amengual JL, Callejas A, et al. (2020) Integrating when and what information in the left parietal lobe allows language rule generalization. PLOS Biology 18(11): e3000895. https://doi.org/10.1371/journal.pbio.3000895

Oliver M. Sawi & Jay Rueckl (2019) Reading and the Neurocognitive Bases of Statistical Learning, Scientific Studies of Reading, 23:1, 8-23, DOI: [10.1080/10888438.2018.1457681](https://doi.org/10.1080/10888438.2018.1457681)

Misyak, J. B., Christiansen, M. H., & Tomblin, J. B. (2010). On-line individual differences in statistical learning predict language processing. *Frontiers in psychology*, *1*, 31. https://doi.org/10.3389/fpsyg.2010.00031

Saffran, J. R., & Wilson, D. P. (2003). From syllables to syntax: Multilevel statistical learning by 12-month-old infants. Infancy, 4(2), 273–284. https://doi.org/10.1207/S15327078IN0402_07

Aslin, Richard & Newport, Elissa. (2012). Statistical Learning: From Acquiring Specific Items to Forming General Rules. Current directions in psychological science. 21. 170-176. 10.1177/0963721412436806.

Schapiro, Anna & Turk-Browne, Nicholas & Norman, Kenneth & Botvinick, Matthew. (2015). Statistical learning of temporal community structure in the hippocampus. Hippocampus. 26. 10.1002/hipo.22523.

Karuza, Elisabeth & Emberson, Lauren & Aslin, Richard. (2013). Combining fMRI and Behavioral Measures to Examine the Process of Human Learning. Neurobiology of learning and memory. 109. 10.1016/j.nlm.2013.09.012.

Schapiro, A. C., Turk-Browne, N. B., Norman, K. A., & Botvinick, M. M. (2016). Statistical learning of temporal community structure in the hippocampus. *Hippocampus*, *26*(1), 3–8. https://doi.org/10.1002/hipo.22523

Niv, Y., & Montague, R. P. (2009). Theoretical and Empirical Studies of Learning. Neuroeconomics, 331–351, Elsevier.

Giavazzi M, Daland R, Palminteri S, Peperkamp S, Brugières P, Jacquemot C, Schramm C, Cleret de Langavant L, Bachoud-Lévi AC. The role of the striatum in linguistic selection: Evidence from Huntington's disease and computational modeling. Cortex. 2018 Dec;109:189-204. doi: 10.1016/j.cortex.2018.08.031. Epub 2018 Sep 15. PMID: 30388440.
