# Supplementary material for: Language statistical learning responds to reinforcement learning principles rooted in the striatum
Source: PLoS Biol. 2021 Sep 7;19(9):e3001119. doi: 10.1371/journal.pbio.3001119 (PMC8448350; doi:10.1371/journal.pbio.3001119)
Supplement: Attachment — Submitted filename: Response_to_Reviewers.docx [file pbio.3001119.s017.docx]

------------------------------------------------------------------------

TITLE:

We would like to suggest a more informative/decompressed title: “Language statistical learning responds to reinforcement learning principles rooted in the striatum.”

We would be happy to work with you on an alternative, if you disagree or feel the our proposed title misrepresents your findings.

**Thank you for suggesting this new title, which conveys the main findings of the paper with precision and succinctly. We have changed the title as suggested to ‘*Language statistical learning responds to reinforcement learning principles rooted in the striatum*.’**

------------------------------------------------------------------------

ETHICS STATEMENT:

Please include within your manuscript the ID number of the protocol approved by the Universitat de Barcelona and the ERC ethics scientific office.

**We have added the ID number of the protocol approved by the University of Barcelona and the ERC ethics scientific office in the appropriate section of *Materials and methods* (lines 421-424), which reads:**

***‘The ERC-StG-313841 (TuningLang) protocol was reviewed and monitored by the European Research Council ethics monitoring office, approved by the ethics committee of the Universitat de Barcelona (IRB 00003099), and conducted in accordance with the Declaration of Helsinki.’***

------------------------------------------------------------------------

DATA POLICY:

You may be aware of the PLOS Data Policy, which requires that all data be made available without restriction: <http://journals.plos.org/plosbiology/s/data-availability>. For more information, please also see this editorial: <http://dx.doi.org/10.1371/journal.pbio.1001797>

Note that we do not require all raw data. Rather, we ask for all individual quantitative observations that underlie the data summarized in the figures and results of your paper. For an example see here: <http://www.plosbiology.org/article/info%3Adoi%2F10.1371%2Fjournal.pbio.1001908#s5>

These data can be made available in one of the following forms:

1) Supplementary files (e.g., excel). Please ensure that all data files are uploaded as 'Supporting Information' and are invariably referred to (in the manuscript, figure legends, and the Description field when uploading your files) using the following format verbatim: S1 Data, S2 Data, etc. Multiple panels of a single or even several figures can be included as multiple sheets in one excel file that is saved using exactly the following convention: S1_Data.xlsx (using an underscore).

2) Deposition in a publicly available repository. Please also provide the accession code or a reviewer link so that we may view your data before publication.

Regardless of the method selected, please ensure that you provide the individual numerical values that underlie the summary data displayed in the following figure panels: Figures 2AB, 3AB, 4, 5, S1AB, S2, S3, S4, and S1 Text.

NOTE: the numerical data provided should include all replicates AND the way in which the plotted mean and errors were derived (it should not present only the mean/average values).

Please also ensure that each figure legend in your manuscript includes information on where the underlying data can be found and that your supplemental data file/s has/have a legend.

Please ensure that your Data Statement in the submission system accurately describes where your data can be found.

**In compliance with PLOS Data Policy, we have created excel files with (or links to, in the case of fMRI data) the data corresponding to figures 2AB, 3AB, 4, 5, S1AB, S2, S3, S4, and S1 Text, as requested.**

I**n line with these additions, we have now included, in the appropriate figure legends, where the data to produce the figure can be found. Thus, p.e., the legend for Fig 2 (lines 125 to 133) now additionally reads:**

***'Data used to generate Fig 2 can be found in S1 Data*.',**

**while that for Fig 4 (lines 220 to 226) reads:**

***'Data used to generate Fig 4 can be found in http://identifiers.org/neurovault.collection:10421'*.**

**We have also added, at the end of the manuscript (lines 743-746), legends for the *Supplementary Information* files pertaining to the data files. E.g.:**

***‘S1 Data. Raw data used to create Fig 2.’* (line 743)**

------------------------------------------------------------------------

DATA NOT SHOWN?

- Please note that per journal policy, we do not allow the mention of "data not shown", "personal communication", "manuscript in preparation" or other references to data that is not publicly available or contained within this manuscript. Please either remove mention of these data or provide figures presenting the results and the data underlying the figure(s).

**Not Applicable**

------------------------------------------------------------------------

**In addition, we have made the following small changes:**

- **Specification of the contrast for the new figure introduced in the previous revision (Fig 5) has now been added to the list of contrasts performed in the corresponding Materials and methods section (line 644).**
- **We have added further detail to more precisely explain how the fMRI results are reported in the paper. This detail was inserted both in the results section (lines 206-208) and in the Materials and methods section (lines 649-650), which now read:**

***'We only report results for clusters FWE-corrected at p < 0.001 at the cluster level (p < 0.001 uncorrected at the voxel level; minimum cluster size = 20).’* (lines 206-208)**

***'Group results are reported for clusters at a p < 0.001 FWE-corrected threshold at the cluster level (p < 0.001 uncorrected at the voxel level), with a minimum cluster extent of 20 voxels.'* (lines 649-650)**

- **Funding details are also more appropriately explained in the acknowledgements section, although we are aware that these would eventually go in the 'Funding' section of the article's first page, if the article was finally accepted for publication.**
- **Finally, we have also reformatted some of the references to better conform to the Plos Biology requirements, corrected a few typos throughout the document (including an error in the examples of the pseudowords used in Fig 1), and improved the printing quality of S1 text figure.**
